# Study of the Impact of Thyroxine Therapy on Bone Turnover Markers in Primary Hypothyroidism: A Cross-Sectional Comparative Study

**DOI:** 10.7759/cureus.112830

**Published:** 2026-07-16

**Authors:** Sunil B Shinde, Anup S Hendre, Axita C Vani

**Affiliations:** 1 Biochemistry, Krishna Institute of Medical Sciences Deemed to be University, Karad, IND

**Keywords:** bone turnover markers, hypothyroidism, levothyroxine, osteoporosis, thyroid function

## Abstract

Introduction

Hypothyroidism affects skeletal metabolism. Thyroid hormones regulate bone remodeling, and their deficiency may alter bone turnover markers (BTMs), increasing the risk of osteoporosis. Levothyroxine therapy restores euthyroid status, but its impact on bone metabolism requires further evaluation.

Aim

This study aims to evaluate the effect of levothyroxine therapy in hypothyroidism on thyroid function and bone turnover markers (C-terminal telopeptide of type I collagen (CTX) and procollagen type I N-terminal propeptide (P1NP)).

Methods

This cross‑sectional comparative study included 150 participants who were further categorized into three groups: Group I (treated hypothyroidism, n=50), Group II (newly diagnosed untreated hypothyroidism, n=50), and Group III (healthy controls, n=50). Baseline clinical data, thyroid function tests (thyroid-stimulating hormone (TSH), total thyroxine (T4), and total triiodothyronine (T3)), and bone turnover markers (serum CTX and P1NP) were measured. For statistical evaluation, chi‑square and one‑way analysis of variance (ANOVA) tests were used, with p<0.05 considered significant.

Results

Group II showed significantly elevated TSH (12.8±4.6 mIU/mL) with reduced T4 and T3 compared to Groups I and III (p<0.001). Serum CTX was highest in Group II (0.48±0.12 ng/mL) versus Group I (0.32±0.08 ng/mL) and Group III (0.28±0.07 ng/mL) (p<0.01). Similarly, P1NP was elevated in Group II (62±15 ng/mL) compared to Group I (45±12 ng/mL) and Group III (40±10 ng/mL) (p<0.01).

Conclusion

Untreated hypothyroidism is found to be linked to more bone resorption and compensatory bone formation, while long‑term levothyroxine therapy normalizes thyroid function and stabilizes bone turnover markers. Early treatment and monitoring of BTMs may help prevent skeletal complications in hypothyroid patients.

## Introduction

Hypothyroidism is a common endocrine disorder characterized by deficient thyroid hormone secretion, which fundamentally impacts body-wide metabolic and physiological pathways. Beyond its classic systemic manifestations, thyroid hormone deficiency severely alters skeletal physiology. Thyroid hormones (thyroxine or T4 and triiodothyronine or T3) are crucial regulators of skeletal development, structural maintenance, and adult bone remodeling. In euthyroid states, these hormones coordinate the balance between osteoclastic bone resorption and osteoblastic bone formation. However, overt hypothyroidism shifts this dynamic, typically suppressing bone turnover and inducing a prolonged bone remodeling cycle characterized by concurrent drops in both osteoclastic and osteoblastic activities. Conversely, some clinical instances point to compensatory fluctuations or distinct structural elevations during varying phases of thyroid failure, emphasizing the highly unpredictable nature of untreated thyroid disease on skeletal architecture [1,2].

A lack of thyroid hormone secretion has been linked to altered bone turnover. Thyroid hormones are essential for skeletal growth and bone remodeling [1]. Bone turnover markers (BTMs), such as procollagen type I N-terminal propeptide (P1NP), a marker of bone production, and C-terminal telopeptide of type I collagen (CTX), a marker of bone resorption, are commonly recognized as reliable indicators of bone metabolic activity [2]. Increased bone remodeling is indicated by elevated levels of these markers, which may put people at risk for fracture and decreased bone mineral density (BMD) [3].

Thyroid impairment has been shown in numerous investigations to affect bone metabolism. Hypothyroidism may result in increased bone resorption and compensatory bone, causing faster bone turnover formation [4]. Significantly, levothyroxine medication, the conventional treatment for hypothyroidism, may correct bone turnover indicators and restore the euthyroid state, which would decrease skeletal risk [5]. CTX and P1NP are suggested as reference markers for assessing bone turnover in clinical and research settings in recent consensus reports from the International Osteoporosis Foundation (IOF) and the International Federation of Clinical Chemistry (IFCC) [2]. IOF and IFCC recommend procollagen type I N-terminal propeptide (P1NP) as the reference marker for bone formation and C-terminal telopeptide of type I collagen (CTX) as the reference marker for bone resorption. Measuring blood-based CTX and P1NP levels delivers a dynamic snapshot of bone remodeling speeds much faster than traditional bone mineral density (BMD) imaging scans like dual-energy X-ray absorptiometry (DEXA). Marked elevations or imbalances in these markers signal accelerated, disorganized remodeling, which impairs bone quality and increases long-term fracture risks. Additionally, new research indicates that thyroid hormone levels and BTMs have a link, underscoring the significance of tracking both indicators in thyroid disorders [6]. In light of these factors, the current study was created to compare thyroid function with bone markers in three groups: healthy controls, just diagnosed and untreated cases, and primary hypothyroidism cases receiving long-term levothyroxine medication. This study attempts to clarify the effects of hypothyroidism treatment on bone metabolism by examining CTX and P1NP levels.

## Materials and methods

This was a cross-sectional comparative observational study conducted in the Department of Biochemistry, Krishna Institute of Medical Sciences, Krishna Vishwa Vidyapeeth Deemed to be University, Karad, Maharashtra, India.

Study participants

Subjects were enrolled from the outpatient and inpatient departments of medicine. As per the inclusion and exclusion criteria, 150 participants were involved in the study. Sample size was calculated based on expected differences in CTX levels between treated and untreated hypothyroid patients, with a power of 80% and a significance level of 5%. The subjects diagnosed with primary hypothyroidism were enrolled and divided into three equal groups: Group I, primary hypothyroidism cases who had been on levothyroxine therapy for at least two years, with a range of 4.9±2.2 years (n=50); Group II, newly diagnosed cases of primary hypothyroidism who were yet to start thyroxine therapy (n=50); and Group III, normal healthy individuals serving as controls (n=50).

Inclusion criteria

Adults aged 18-60 years, those with a confirmed diagnosis of primary hypothyroidism based on elevated thyroid-stimulating hormone (TSH) and low T4 levels, patients on stable levothyroxine therapy for ≥2 years (the duration of levothyroxine therapy was in the range of 4.9±2.2 years) (Group I), newly diagnosed cases with no prior thyroid hormone replacement (Group II), and those willing to provide written informed consent were included.

Exclusion criteria

We excluded those with a history of secondary or central (pituitary/hypothalamic) hypothyroidism; those with known metabolic bone disorders (e.g., osteoporosis, Paget’s disease, and osteomalacia); those with chronic kidney disease (estimated glomerular filtration rate (eGFR) < 60 mL/min/1.73 m²), liver dysfunction, or active malignancy; those who used medications known to affect bone metabolism (e.g., systemic corticosteroids, bisphosphonates, anticonvulsants, and heparin); pregnant women and lactating mothers; those with vitamin D deficiency (<10 ng/mL) requiring active pharmacological supplementation; and those with a history of calcium or phosphate disorders.

All participants underwent a thorough clinical evaluation, including detailed history, physical examination, anthropometric measurements (height, weight, and body mass index (BMI)), and assessment of duration and symptoms of hypothyroidism. Demographic information, such as age and body mass index (BMI), was recorded. BMI was assessed by formula as weight in kilograms divided by height in meters squared (kg/m²). A blood sample was taken following overnight fasting, centrifuged at 3,000 rpm for 10 minutes, and analyzed immediately for all biochemical parameters. Serum thyroid-stimulating hormone (TSH), total thyroxine (T4), and total triiodothyronine (T3) were analyzed using chemiluminescent immunoassay methods. Serum C-terminal telopeptide of type I collagen (CTX) and serum procollagen type I N-terminal pro-peptide (P1NP) were analyzed as bone resorption and bone formation markers, respectively.

Ethical considerations

The study protocol was approved by the Institutional Ethics Committee of Krishna Institute of Medical Sciences Deemed to be University, Karad (approval number: KIMSDU/IEC/04/2023). Written informed consent was obtained from all participants prior to enrollment. Patient confidentiality was maintained throughout the study. The study was conducted in accordance with the Declaration of Helsinki and Good Clinical Practice guidelines.

Statistical analysis

Data were entered into Microsoft Excel (Microsoft Corp., Redmond, WA) and analyzed using SPSS Statistics version 25.0 (IBM Corp., Armonk, NY). The data has been represented as “N” with percentage (%) calculated. Continuous variables were expressed as mean±standard deviation (SD). Intergroup comparisons of continuous variables were performed using the independent samples t-test. Categorical variables were compared using the chi-square test. Pearson correlation coefficients were calculated to assess the relationship between bone turnover markers (CTX) and thyroid function parameters (TSH, total T4, and total T3). A p-value < 0.05 was considered statistically significant. All tests were two-tailed.

## Results

Table 1 presents a baseline clinical profile comparing the three study groups (Group I, II, and III), each consisting of 50 participants, evaluated across age and body mass index (BMI) categories. The majority of participants across all groups fall in the 31-60 years category, representing 31 (62%) in Group I, 29 (58%) in Group II, and 27 (54%) in Group III. The derived p-value is 0.96. This value is well above the standard significance threshold (p>0.05). This indicates that there is no statistically significant difference in age distribution among the three groups, rendering them well-matched and comparable regarding age.

**Table 1:** Baseline Clinical Profile of the Study Participants BMI: body mass index

Serial number		Group I (n=50)	Group II (n=50)	Group III (n=50)	p-value
1	Age (years) ≤ 30	9 (18%)	11 (22%)	13 (26%)	0.96 (non-significant)
	31-60	31 (62%)	29 (58%)	27 (54%)	
	>60	10 (20%)	10 (20%)	10 (20%)	
2	BMI (kg/m²) < 18	3 (6%)	4 (8%)	5 (10%)	0.07 (non-significant)
	18-24	21 (42%)	19 (38%)	30 (60%)	
	>24	26 (52%)	27 (54%)	15 (30%)	

Group I and Group II present higher proportions of overweight individuals with a BMI > 24 kg/m² (n=26 (52%) and n=27 (54%), respectively). In contrast, Group III shows a predominantly normal BMI distribution between 18 and 24 kg/m² (n=30 (60%)). The derived p-value is 0.07. Because this value is greater than 0.05, the variation in BMI across the cohorts is not statistically significant. However, it is often described as approaching or “close to” statistical significance, suggesting a notable trend toward lower BMIs in Group III.

Table 2 shows the levels of thyroid function test parameters across the three studied cohorts, demonstrating highly significant variations among the groups. Serum thyroid-stimulating hormone (TSH) levels are markedly elevated in Group II (newly diagnosed hypothyroidism) at 12.8±4.6 mIU/mL compared to Group I (treated hypothyroidism) at 3.2±1.5mIU/mL and Group III (healthy controls) at 2.4±1.2mIU/mL. Conversely, thyroid hormone concentrations are significantly depressed in the untreated Group II cohort. Total thyroxine (T4) levels drop to 4.2±1.5 µg/dL in Group II, whereas Group I (8.5±2.1 µg/dL) and Group III (9.1±2.0 µg/dL) maintain comparable normal-range values. Similarly, total triiodothyronine (T3) is markedly reduced in Group II (70±20 ng/mL) compared to Group I (110±30 ng/mL) and Group III (120±30 ng/mL). Across all three parameters (TSH, total T4, and total T3), the calculated p-values are uniform (<0.001), establishing a highly statistically significant difference that confirms severe thyroid hormone deficiency and compensatory TSH elevation in the newly diagnosed, untreated hypothyroid group.

**Table 2:** Levels of Thyroid Function Test Parameters Among the Groups TSH: thyroid-stimulating hormone, T4: total thyroxine, T3: total triiodothyronine, SD: standard deviation

Serial number	Parameter	Group I (n=50)	Group II (n=50)	Group III (n=50)	F-value	p-value
1	TSH (mIU/mL), mean±SD	3.2±1.5	12.8±4.6	2.4±1.2	202.17	<0.001 (significant)
2	Total T4 (µg/dL), mean±SD	8.5±2.1	4.2±1.5	9.1±2.0	100.52	<0.001 (significant)
3	Total T3 (ng/mL), mean±SD	110±30	70±20	120±30	47.73	<0.001 (significant)

Table 3 illustrates the comparison of serum C-terminal telopeptide (CTX) levels among the three study cohorts, revealing a statistically significant variation across the groups. The highest mean concentration of serum CTX is observed in Group II (newly diagnosed hypothyroidism) at 0.48±0.12 ng/mL. In comparison, Group I (treated hypothyroidism) exhibits a lower mean level of 0.32±0.08 ng/mL, while Group III (healthy controls) displays the lowest baseline level at 0.28±0.07 ng/mL. The calculated p-value for this comparison is <0.01, indicating that the differences in bone resorption markers across the three groups are highly statistically significant. This suggests that untreated hypothyroidism is associated with an alteration in bone turnover (elevated CTX), which appears to trend back toward control levels following treatment.

**Table 3:** Comparison of Bone Resorption Marker CTX Among the Groups CTX: C-terminal telopeptide of type I collagen, SD: standard deviation

Serial number	Serum CTX (ng/mL)	Group I (n=50)	Group II (n=50)	Group III (n=50)	F-value	p-value
1	Mean	0.32	0.48	0.28	55.93	<0. 01 (significant)
2	±SD	0.08	0.12	0.07		

**Figure 1:**
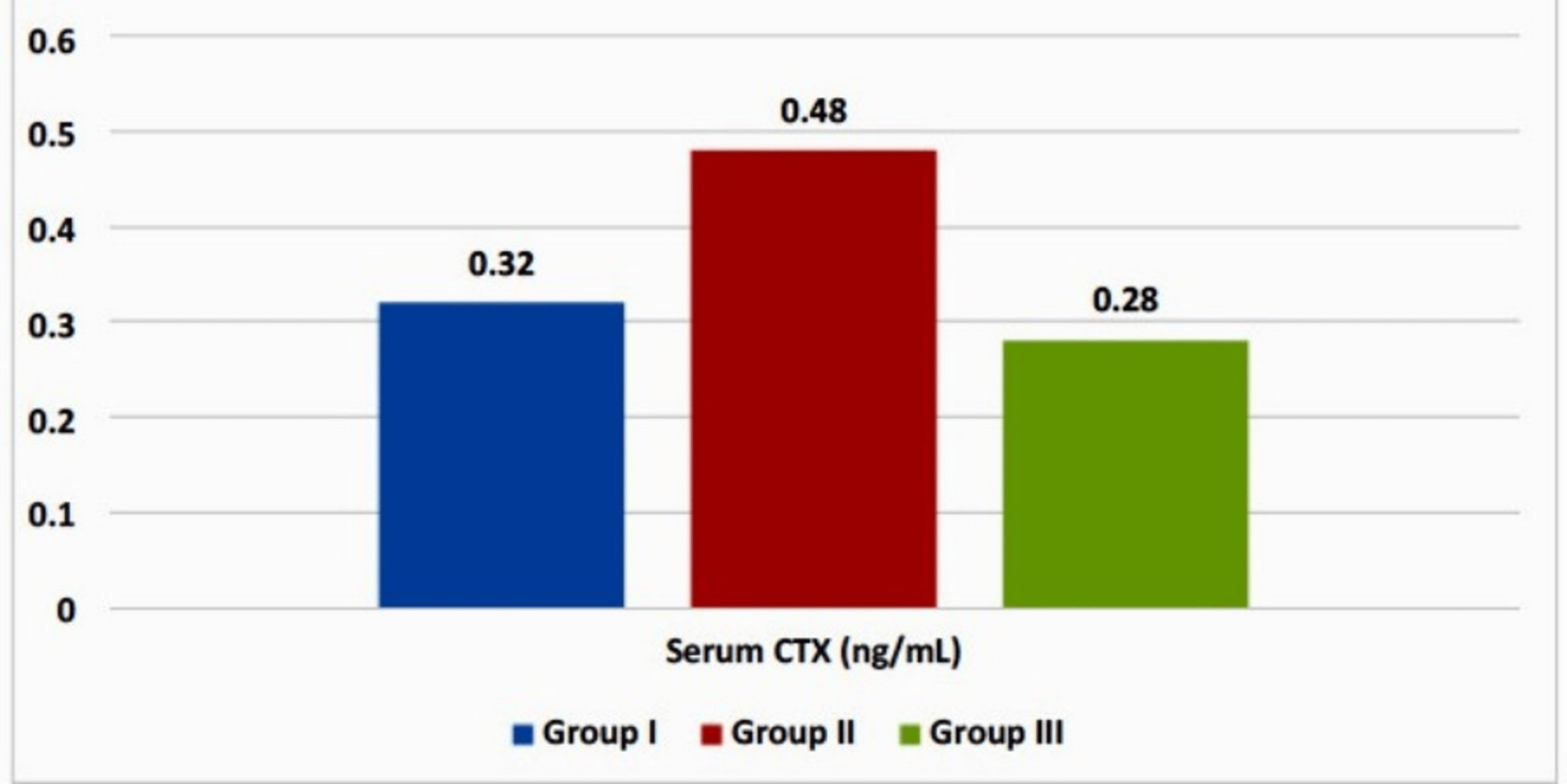
Comparison of Bone Resorption Marker CTX Among the Groups CTX: C-terminal telopeptide of type I collagen

Figure 1 compares the levels of the bone resorption marker serum C-terminal telopeptide of type I collagen (CTX) among the three study groups. Newly diagnosed, untreated primary hypothyroidism cases (Group II) exhibit the highest serum CTX concentration at 0.48 ng/mL. Patients with primary hypothyroidism who have undergone levothyroxine therapy for at least two years (Group I) present intermediate levels at 0.32 ng/mL. Healthy control individuals (Group III) show the lowest baseline serum CTX levels at 0.28 ng/mL. According to these results, bone resorption activity is significantly higher in untreated hypothyroidism but trends back down toward normal control values with long-term levothyroxine replacement therapy.

Table 4 details the comparison of the bone formation marker procollagen type I N-terminal propeptide (P1NP) among the three study cohorts, demonstrating a statistically significant difference across the groups. The mean serum P1NP concentration is highest in Group II (newly diagnosed and untreated hypothyroidism) at 62±15 ng/mL. This is followed by Group I (treated hypothyroidism) with a mean level of 45±12 ng/mL, while Group III (healthy controls) exhibits the lowest mean baseline level at 40±10 ng/mL. The derived p-value for this comparison is p<0.01, indicating that the variations in bone formation activity among the cohorts are highly statistically significant. These findings show that bone turnover markers, specifically bone formation via P1NP, are significantly altered in untreated hypothyroidism and trend closer to healthy control values following thyroid treatment.

**Table 4:** Comparison of Bone Formation Marker P1NP Among the Groups P1NP: procollagen type I N-terminal propeptide, SD: standard deviation

Serial number	Serum P1NP (ng/mL)	Group I (n=50)	Group II (n=50)	Group III (n=50)	F-value	p-value
1	Mean	45	62	40	42.54	<0.01 (significant)
2	±SD	12	15	10		

**Figure 2:**
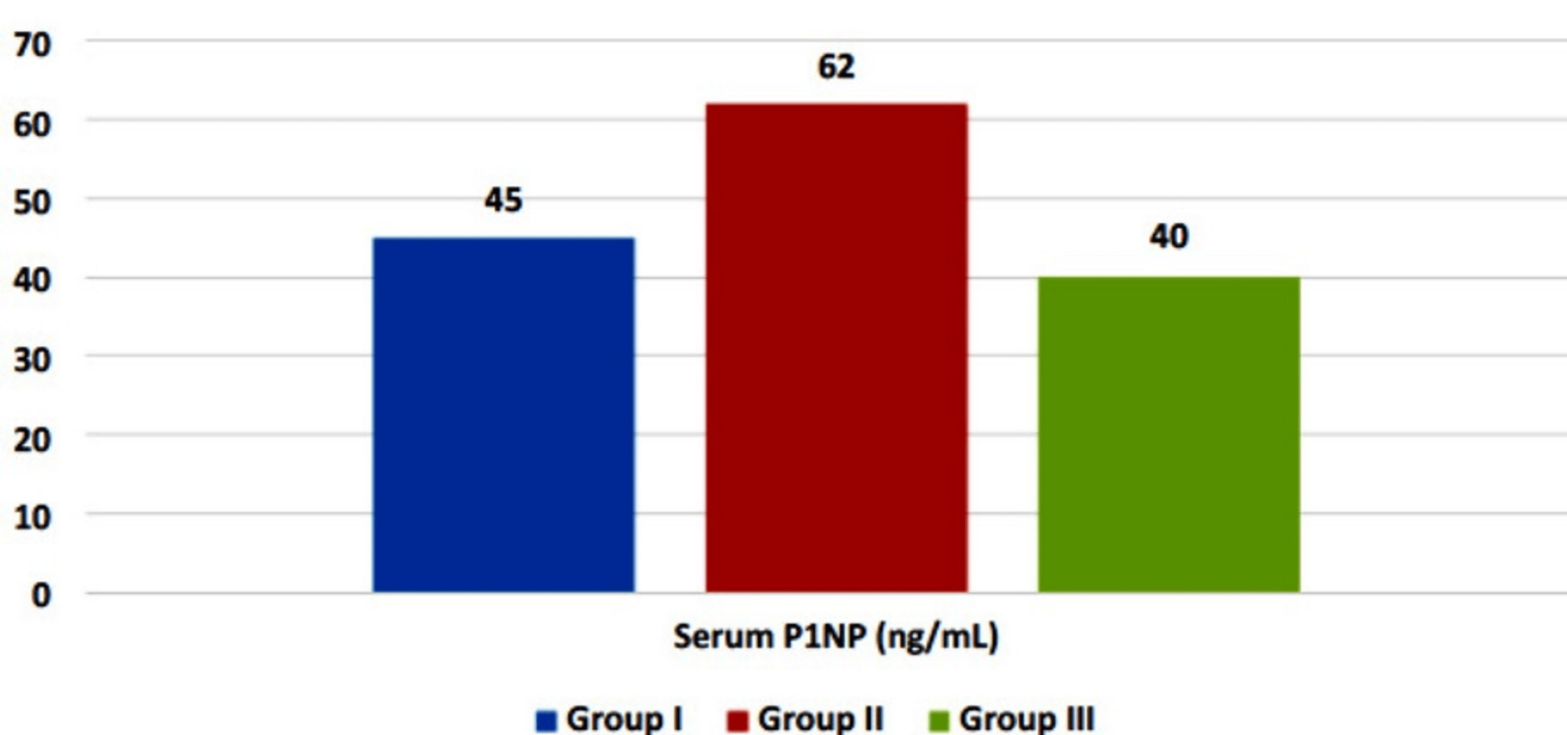
Comparison of Bone Formation Marker P1NP Among the Groups P1NP: procollagen type I N-terminal propeptide

Figure 2 compares the concentrations of the bone formation marker serum procollagen type I N-terminal propeptide (P1NP) across the three study groups. Patients with newly diagnosed, untreated primary hypothyroidism (Group II) have the highest blood P1NP level (62 ng/mL). The intermediate value of 45 ng/mL is seen in people with primary hypothyroidism who have been receiving stable levothyroxine medication for at least two years (Group I). At 40 ng/mL, the baseline concentration of the normal, healthy persons acting as controls (Group III) is the lowest. This distribution shows that bone creation tracking follows an elevated bone turnover rate in untreated hypothyroid situations, which moderately downregulates toward normal baseline values following long-term thyroxine replacement therapy. This pattern is comparable to that of the bone resorption marker.

## Discussion

Newly diagnosed and untreated cases (Group II) showed higher TSH levels with lower T4 and T3 compared to treated patients and healthy controls. These results mimicked previous study findings that levothyroxine medication restores almost normal thyroid hormone levels while untreated hypothyroidism is characterized by high TSH and decreased thyroid hormone values [7,8].

There were clear differences in bone turnover markers between the groups. Untreated hypothyroid cases showed higher levels of serum CTX, a marker of bone resorption, than both treated patients and controls. This is consistent with the research by Su et al. that found elevated bone resorption activity in thyroid disease, especially in untreated states [3]. Similarly, Yoo et al. found higher levels of bone turnover markers in endocrine disorders and correlated them with thyroid hormone status [5]. The normalization of CTX levels in patients receiving long-term levothyroxine medication indicates that sufficient replacement reduces skeletal risk by moderating excessive bone resorption.

In comparison to treated patients and controls, untreated cases showed higher levels of serum P1NP, a marker of bone production. This result is consistent with that of Mengxue et al., who observed compensatory increases in bone formation markers in hypothyroid conditions, which may represent a homeostatic reaction to elevated resorption [4]. The reduction of P1NP levels in treated patients toward control values indicates that levothyroxine therapy stabilizes bone remodeling.

The direct impact of thyroid hormones on osteoblast and osteoclast activity could be the mechanism underlying these findings. By promoting osteoblastic differentiation and indirectly increasing osteoclastic bone, thyroid hormones control skeletal remodeling resorption [2,8]. Reduced thyroid hormone availability in hypothyroidism causes aberrant signaling, which in turn causes an imbalance in bone turnover.

In untreated patients, elevated CTX and P1NP may indicate a dysregulated remodeling process with compensatory formation and enhanced resorption. Levothyroxine restores the euthyroid state, which stabilizes bone by restoring these metabolic pathways. As bone resorption markers such as CTX surge, the skeleton deploys an early homeostatic defense, driving up bone production markers such as P1NP to patch the structural voids left by hyper-resorption. This dynamic matches a 2025 meta-analysis by Li et al., which observed that early-stage untreated hypothyroidism often prompts unexpected, compensatory spikes in biochemical markers of bone turnover [5,9-11].

Crucially, Group III (long-term levothyroxine-treated patients) demonstrated near-complete normalization of both CTX and P1NP, verifying that restoring a euthyroid state restabilizes these unbalanced bone pathways. A recent 2026 clinical cohort study by Vasamsetti et al. confirmed that targeted levothyroxine replacement significantly mitigates skeletal fragility and optimizes systemic biochemical parameters within months of restoring a euthyroid window [12].

Overall, the present findings support earlier research and emphasize how crucial thyroid hormone replacement is to preserving bone health. The metabolic effects of hypothyroidism and the efficacy of treatment can be better understood by tracking bone turnover indicators such as CTX and P1NP.

The study compares three separate cohorts rather than following the same untreated group (Group II) longitudinally over time. A pre- and post-treatment follow-up design would provide stronger evidence of levothyroxine’s direct impact on bone markers. Also, the sample size is relatively small, with 50 participants per group (N=150). This may limit the statistical power to detect or generalize the findings to a larger population. The data appears to be collected from a single institution, which might restrict its applicability to different geographical, ethnic, or demographic backgrounds. Our study has several limitations that warrant consideration when interpreting the findings. Certain lifestyle variables such as smoking habits and alcohol consumption, which can independently alter bone metabolism, were not fully accounted for in our patient cohorts. Furthermore, we lacked detailed records on the use of menopausal hormone therapy among the female participants. Because estrogen status is a critical determinant of bone remodeling rates, the absence of this variable may introduce unmeasured confounding into our analysis. Future prospective trials with strictly controlled matching or comprehensive multivariable adjustment for these lifestyle and metabolic regulators are necessary to validate our observations.

## Conclusions

In conclusion, untreated hypothyroidism in female patients was associated with elevated serum TSH, decreased thyroid hormones, and accelerated bone turnover markers (CTX and P1NP), suggesting uncoupled high bone turnover. Conversely, long-term levothyroxine replacement therapy appears to restore a euthyroid state and stabilize bone metabolism. These findings underscore the clinical relevance of early intervention and suggest that monitoring bone turnover markers may provide valuable, real-time insights into thyroid-induced metabolic shifts in bone health, even before measurable changes in bone mineral density occur.
